# Dual Band and Dual Diversity Four-Element MIMO Dipole for 5G Handsets

**DOI:** 10.3390/s21030767

**Published:** 2021-01-24

**Authors:** Muhammad Ali Jamshed, Masood Ur-Rehman, Jaroslav Frnda, Ayman A. Althuwayb, Ali Nauman, Korhan Cengiz

**Affiliations:** 1 Department of Electrical Engineering, Institute of Space Technology, Islamabad 44000, Pakistan; mohammadalijamshed@gmail.com; 2 James Watt School of Engineering, University of Glasgow, Glasgow G12 8QQ, UK; masood.urrehman@glasgow.ac.uk; 3 Department of Quantitative Methods and Economic Informatics, Faculty of Operation and Economics of Transport and Communications, University of Zilina, 010 26 Zilina, Slovakia; jaroslav.frnda@fpedas.uniza.sk; 4 Electrical Engineering Department, Jouf University, Sakaka, Aljouf 72388, Saudi Arabia; aaalthuwayb@ju.edu.sa; 5 Department of Information and Communication Engineering, Yeungnam University, Gyeongsan-si 38541, Gyeongsangbuk-do, Korea; 6 Department of Electrical-Electronics Engineering, Trakya University, Edirne 22030, Turkey; korhancengiz@trakya.edu.tr

**Keywords:** multiple-input-multiple-output (MIMO), printed dipole, 5G handset antennas, dual band, dual diversity

## Abstract

The increasing popularity of using wireless devices to handle routine tasks has increased the demand for incorporating multiple-input-multiple-output (MIMO) technology to utilize limited bandwidth efficiently. The presence of comparatively large space at the base station (BS) makes it straightforward to exploit the MIMO technology’s useful properties. From a mobile handset point of view, and limited space at the mobile handset, complex procedures are required to increase the number of active antenna elements. In this paper, to address such type of issues, a four-element MIMO dual band, dual diversity, dipole antenna has been proposed for 5G-enabled handsets. The proposed antenna design relies on space diversity as well as pattern diversity to provide an acceptable MIMO performance. The proposed dipole antenna simultaneously operates at 3.6 and 4.7 sub-6 GHz bands. The usefulness of the proposed 4×4 MIMO dipole antenna has been verified by comparing the simulated and measured results using a fabricated version of the proposed antenna. A specific absorption rate (SAR) analysis has been carried out using CST Voxel (a heterogeneous biological human head) model, which shows maximum SAR value for 10 g of head tissue is well below the permitted value of 2.0 W/kg. The total efficiency of each antenna element in this structure is −2.88, −3.12, −1.92 and −2.45 dB at 3.6 GHz, while at 4.7 GHz are −1.61, −2.19, −1.72 and −1.18 dB respectively. The isolation, envelope correlation coefficient (ECC) between the adjacent ports and the loss in capacity is below the standard margin, making the structure appropriate for MIMO applications. The effect of handgrip and the housing box on the total antenna efficiency is analyzed, and only 5% variation is observed, which results from careful placement of antenna elements.

## 1. Introduction

The advancements in the wireless communication industry and, more specifically, the fifth generation (5G) of mobile communication technology have entirely shifted how many societies communicate and transfer information. This shift has raised the need to explore technologies, which can support this immense amount of data transfer by using the wireless communication systems. The antennas relying on multiple-input-multiple-output (MIMO) technology have enabled 5G to meet the latency, capacity, and the bandwidth requirements to support the demands of the ever-growing number of wireless communication users [1,2]. A lot of research efforts have been put to increase the number of active antenna elements on the base station (BS) to enhance the overall system capacity [3] and increase bandwidth utilization. For instance, in [4] the challenges and the opportunities in developing the MIMO antennas for the BSs, supporting the future wireless communication systems, are explained. In [5] a dual-polarized 2 × 4, four-element MIMO antenna for 5G-enabled BSs is proposed, and the authors use magnetic conductors to achieve required isolation for MIMO systems. A mechanism based on vector synthesis is used in [6] to minimize the cross-polarization as much as possible and simultaneously increase the port-to-port isolation to meet the MIMO criteria. Similarly, in [7], Fylfot-shaped MIMO antenna operating at 0.7 GHz to 0.96 GHz as well as 1.7 GHz to 2.7 GHz for 5G-enabled BS is proposed, and the MIMO performance is meet using the mechanism of overlapping the sub-arrays, which is also verified using the basic MIMO parameters. Moreover, a four-element U-shaped low profile MIMO antenna design, covering a significant portion of LTE as well as the new 5G bands is proposed in [8]. It shows that the isolation and the envelope correlation coefficient (ECC) required for MIMO applications is satisfied.

Although the MIMO’s usefulness or the massive MIMO can easily be exploited at the BS [9], the size limitations at the mobile handsets or the user terminal (UT), impose additional challenges. These challenges limit the space required to accommodate the increasing number of antenna elements to support the MIMO operation. For instance, in [10], a 2 × 2 MIMO antenna structure is proposed for long term evolution (LTE) enabled handsets, which uses the space diversity to achieve isolation of −15 dB. In [11], a four-element MIMO monopole is proposed that first uses the concept of space diversity. A microstrip tapered fed-line is further incorporated in the structure to achieve an ECC of 0.014 and isolation of −21 dB, between each antenna pair, making them practical for MIMO systems. In [12], the authors have relied on the space diversity to propose a four-element 2 × 2 MIMO rim antenna operational at LTE bands. They have shown their design’s effectiveness through the liquid crystal display (LCD) and the human handgrip analysis. Similarly, in [13], the authors use the metallic rim to propose a classic 2 × 2 MIMO rim antenna for LTE bands, while mainly using the space diversity, hence making their design simple and effective. Moreover, the pattern reconfiguration antennas arranged in a MIMO configuration, suitable for state-of-the-art mobile handsets has been proposed in [14]. However, it is a tedious task to achieve the required isolation, ECC and the loss in capacity, to maintain the MIMO efficiency by mainly relying on the use of space diversity for tightly packed antenna elements.

In this paper, rather than just relying on space diversity, we have also incorporated the radiation pattern diversity, in our proposed antenna design. By combining both radiation pattern and space diversity features, we have proposed a novel 4 × 4 dual-band, dual diversity, dipole antenna suitable for 5G-enabled handsets. The proposed 4 × 4 dual-band, dual diversity, dipole antenna has been fabricated, and measured results are compared with the simulated. The current distribution plots validate the dual-band and the dual diversity operation. Moreover, by performing a specific absorption rate (SAR) analysis, we have shown our design’s practicality for real-life applications. The MIMO performance analysis has shown the suitability of our proposed design for the MIMO-enabled state-of-the-art mobile handsets. Furthermore, we have adjusted each element’s placement so that the effect of the handgrip and the housing box on the total antenna efficiency is as minimal as possible.

The rest of the paper is organized as follows: Section 2, of the paper, provides an in-detail discussion on the related literature and compares the proposed design with the state-of-the-art. Section 3 of the paper illustrates the dimensions and the structure of the proposed antenna design using multiple views. This Section also explains the different materials used to replicate the housing box, incorporated in our antenna structure to test the variation in total antenna efficiency. In Section 4, we have performed a study to show our proposed antenna design’s effectiveness when used for MIMO systems. Section 4 shows a SAR analysis to validate our design’s practicality and studies the housing box’s effect and the human handgrip on the total antenna efficiency. In Section 5, we conclude the paper.

## 2. Discussion on Related Works

The current state-of-the-art mobile handsets are equipped with additional features and complex circuitry to provide ease to the end-users in handling routine tasks. These additive features require an increased amount of electronic circuitry, which leaves minimal space for the antennas to be placed on the mobile handset. Most of the work available in the literature rely on space diversity and uses various complex techniques to achieve the minimum requirement set for the MIMO operation, i.e., −15 dB of isolation, ECC of 0.3, and a loss in capacity of less than 0.4 bits per second (bps), over the desired bandwidth [15]. Limited space on the UTs leads to complex antenna structures for the fulfilment of these requirements. For instance, in [16], the authors use a T-shaped radiator to enhance the isolation between closely spaced antenna elements and to enable them to operate as a MIMO.

In [17], two vertical stubs are introduced to enhance the isolation between antenna elements used for MIMO-based mobile handsets. In [18], an out of band parasitic element approach is used to maintain constant isolation of −20 dB, between each antenna pair. Similarly, in [19], the reverse coupling has been created using the monopole-based parasitic elements to reduce the amount of mutual coupling between the antenna element pair, hence making them suitable for MIMO-enabled handsets. Moreover, the concept of using the meander lines is applied between two closely spaced microstrip MIMO antenna elements to reduce the isolation [20] and to achieve the required ECC values. Furthermore, the mutual coupling is reduced to −24.7 dB by employing three interdigital lines between the two patch antenna elements by creating orthogonal polarization modes [21], making them practical for MIMO applications.

A dual 2 × 2 MIMO operating at sub-6 GHz band and a 4 × 4 MIMO operating at mmWave band are proposed in [22], which incorporates the defects in the ground plane to achieve the required isolation to maintain a suitable MIMO efficiency by maintaining the mutual coupling below −15 dB. A Layering concept using via-holes is proposed in [23], which can reduce each antenna element’s overall size by 50%, hence providing enough degree of freedom to increase the number of antenna elements on a mobile handset. The use of neutralization lines is another technique that can be employed between the antenna elements, hence enabling them to work as a MIMO [24,25]. In [26], a similar concept of neutralization lines have been used to create decoupling between closely packed ultra wide-band MIMO antennas. Instead of just relying on the concept of using the neutralization lines, the use of slots on the ground place can also be employed between antenna elements to enhance the isolation and make them suitable for the MIMO systems [27]. Table 1 we have shown the comparison of our proposed work with the relevant literature in terms of space and the radiation pattern diversity used to achieve the required MIMO performance.

## 3. Antenna Design

**Figure 1:**
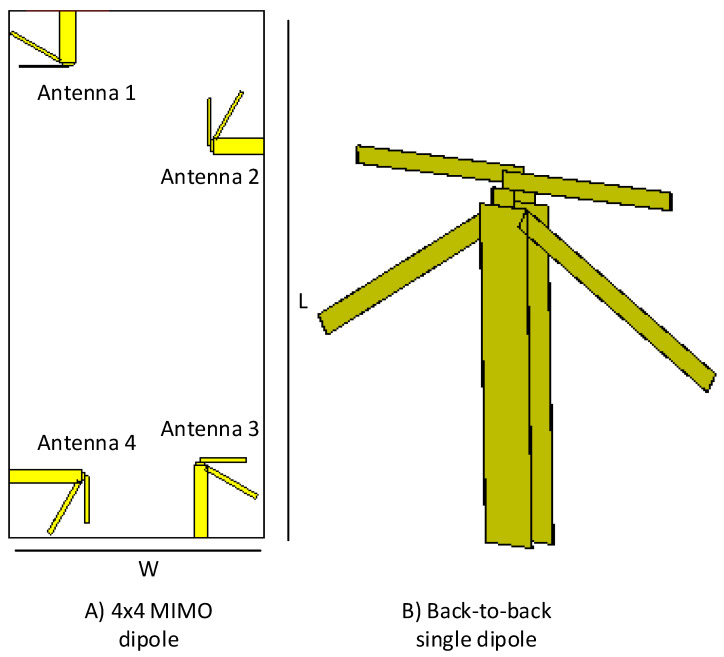
(**A**) Dimensions/size of the substrate used for the proposed dual-band dual diversity four-element MIMO antennas design, (**B**) 3D/back-to-back view of the proposed dipole antenna.

In this section, we illustrate the structure of our proposed antenna design by using multiple views. The substrate’s dimensions, placement of each of the antenna element present in the structure, and the 3D/back-to-back view of a single antenna element of the proposed dipole design are shown in Figure 1. Figure 1A shows the dimensions of the substrate used, which is equivalent to the size of any standard smartphone available in the market. Length *L* of the substrate is set as L=138 mm, and the width *W* of the substrate is chosen to be W=67 mm. Rogers 5880 has been used as a substrate material to support the radiating antenna elements. The dielectric constant, $\epsilon_{r}$ of the substrate material is $\epsilon_{r}=2.2$ having a dissipation factor tanδ of 0.0005. The substrate thickness is 1.6 mm, whereas the thickness of copper (double-sided) is set as 35 μm. Figure 1B shows a 3D/back-to-back view of a single dipole antenna element with a balance feed-line, which acts as a balun to feed the proposed printed dipole antennas.

**Figure 2:**
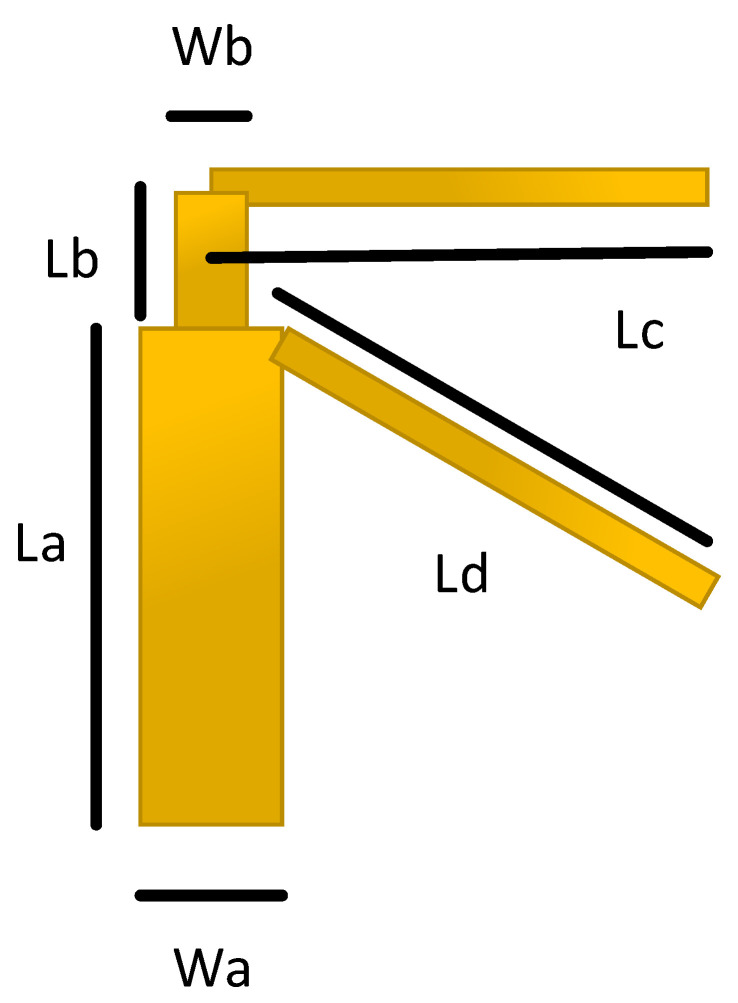
Dimensions of a single proposed dual band dipole antenna design.

**Figure 3:**
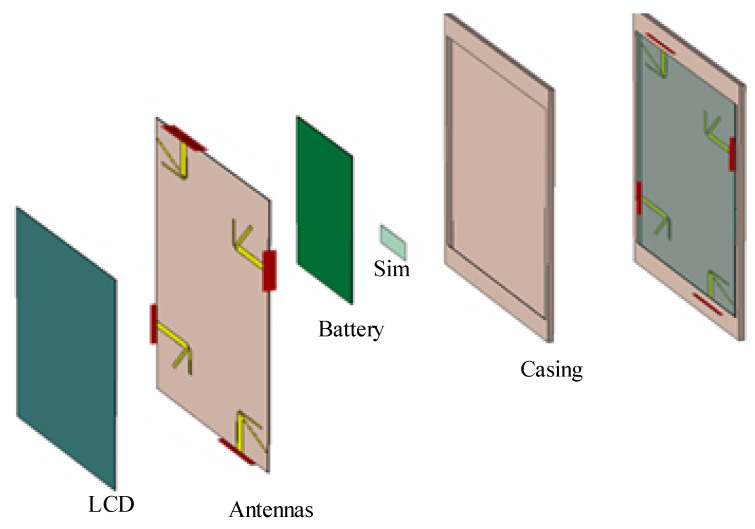
Composition of the housing box used.

Figure 2, displays the dimensions of a single dipole antenna element, where the values of Ld and Lc are ≈0.48 λ, and La is ≈λ/4, for each antenna element. The values of Wa, Wb, and Lb are adjusted using detailed parametric analysis, performed in Computer Simulation Technology (CST) Studio Suite^®^ 2018. The values of these design parameters are further listed in Table 2. The Figure 3, depicts the hierarchical cross-section view of the housing box being used to test the stability of the proposed antenna design for the real environment. The housing box is made up of a battery, LCD, casing, and a subscriber identity module (SIM). The housing box battery is composed of nickel, having a thermal conductivity of 91 W/K/m, and electrical conductivity of $1.44\times 10^{7}$ S/m. Whereas the LCD of the housing box is composed of lead glass, with electrical conductivity of $1\times 10^{-12}$ S/m, the casing is composed of polycarbonate thermal conductivity of 0.19 W/K/m. The material used for the SIM is silicon, with a thermal conductivity of 198 W/K/m.

## 4. Performance Analysis

**Figure 4:**
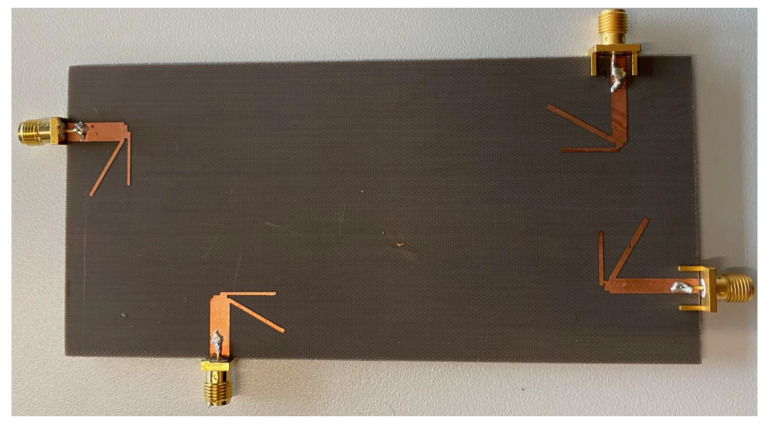
Prototype of the proposed antenna design.

**Figure 5:**
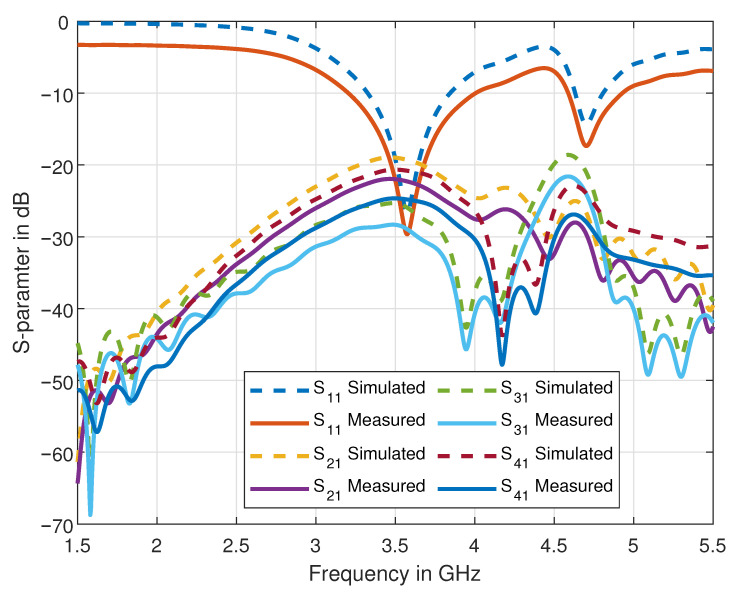
Comparison of simulated and measured S-parameters of the proposed antenna design.

The prototype of our novel four-element dipole 4 × 4 MIMO antenna design is fabricated and is shown in Figure 4. The performance of the proposed antenna design is confirmed by comparing the simulated results with the measured results. Figure 5 shows a comparison of simulated and measured S-parameters of the proposed dipole antennas. Since our design is symmetrical, we have shown the results of a single antenna element only, i.e., Antenna 1 as labelled in Figure 1A. The measured and simulated $S_{11}$ for Antenna 1 (because of symmetry other elements would have similar results), is well below −10 dB and isolation between elements, i.e., for instance, $S_{21}$, $S_{31}$, and $S_{41}$, are below −15 dB. Since each antenna element’s isolation is below −15 dB, the proposed antenna can be considered an excellent candidate to satisfy the MIMO criteria.

**Figure 6:**
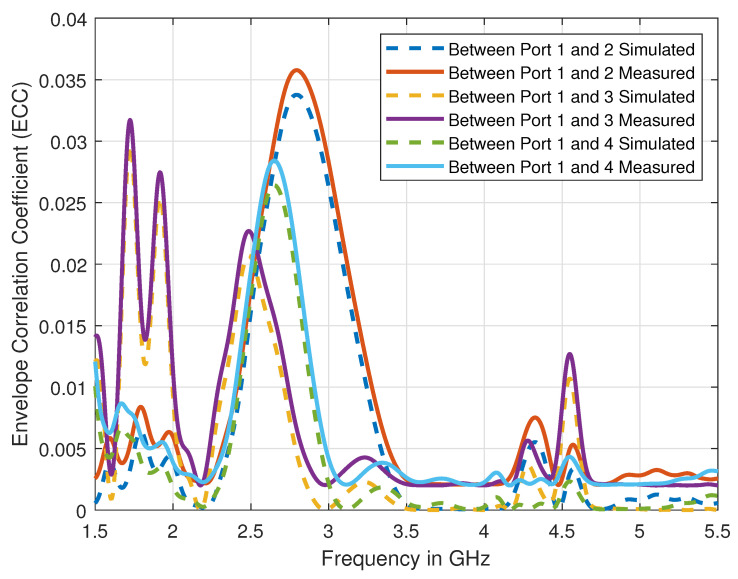
Comparison of simulated and measured ECC of the proposed antenna design.

To validate the proposed antenna’s suitability for MIMO applications, we have calculated the ECC and the loss in capacity by using the measured and the simulated S-parameters results. The Figure 6 shows the variation of simulated ECC over the defined range of frequencies. The values of ECC over the operating resonance frequencies, i.e., 3.6 GHz and 4.7 GHz, are well below 0.005, which indeed verify the suitability of our proposed antenna design for MIMO applications. The loss in capacity for the two-element antenna system by using the reflection coefficients can be expressed as [28]:

(1) $$\begin{array}{c} {\bar{C}}_{loss}=-\log_{2}\left(\det\left(\gamma^{r}\right)\right). \end{array}$$

Using the measured S-parameters data as shown in Figure 5, the value of loss in capacity is calculated to be ≈0.19 bps at 3.6 GHz and ≈0.18 at 4.7 GHz, which is lower than the required threshold. Indeed, ECC’s calculated values and the loss in capacity show that our proposed antenna design can ensure an acceptable MIMO performance.

**Figure 7:**
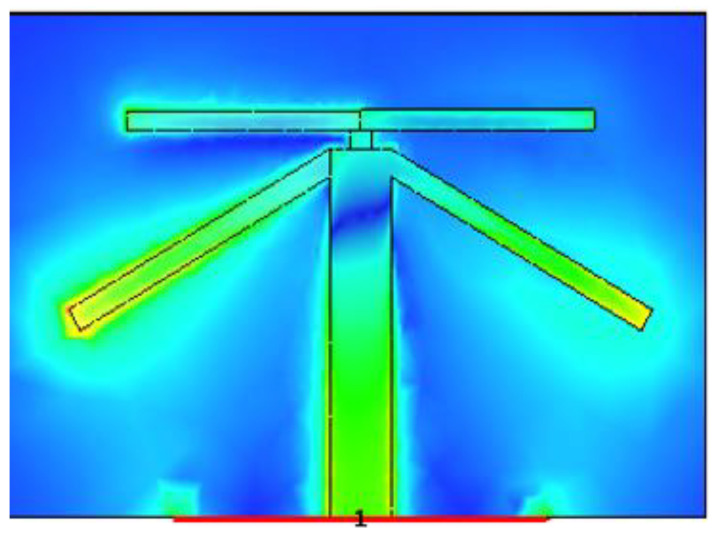
Illustration of the current distribution at 3.6 GHz.

**Figure 8:**
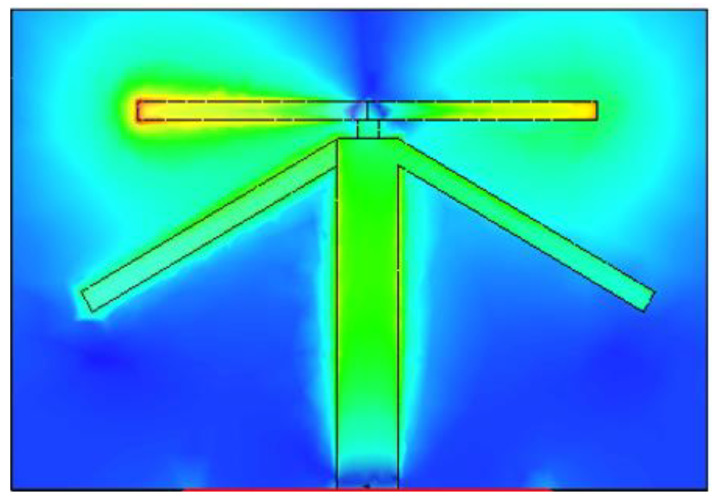
Illustration of the current distribution at 4.7 GHz.

**Figure 9:**
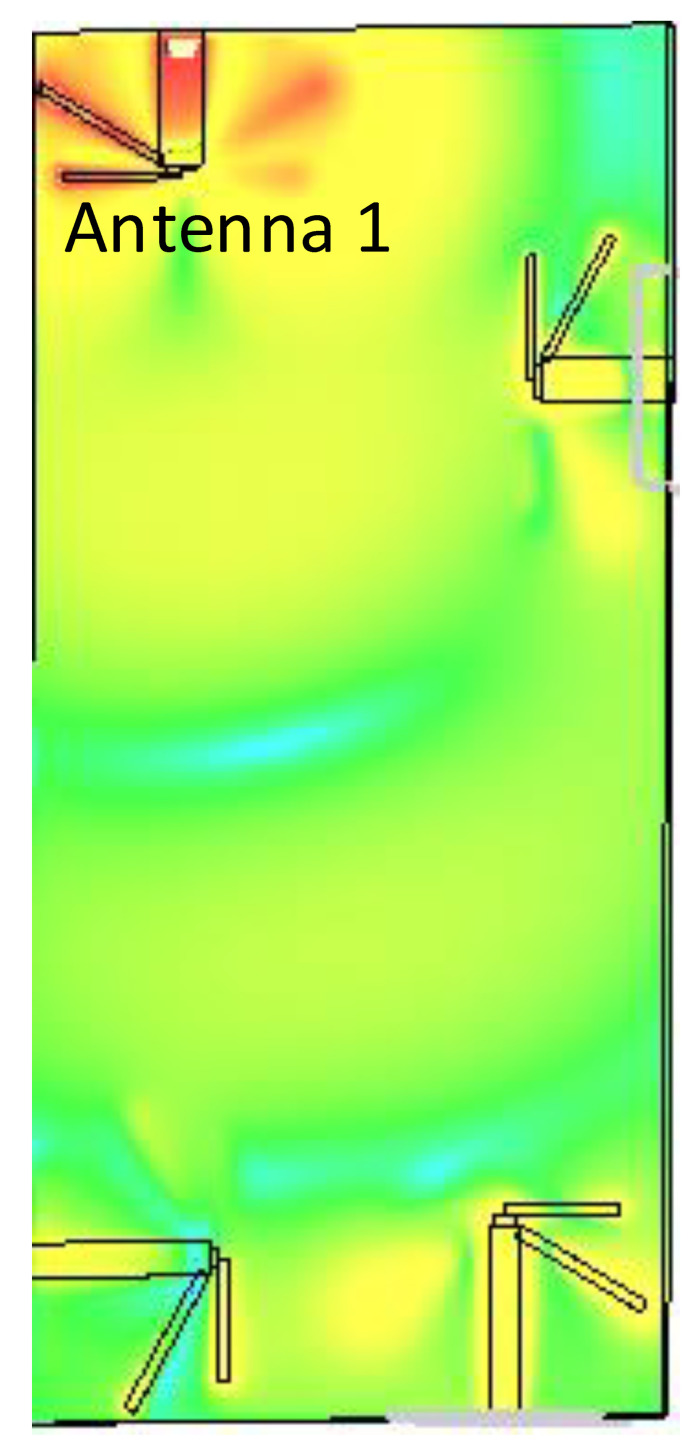
Current distribution at 3.6 GHz for Antenna 1 in comparison to other elements.

Figure 7, Figure 8 and Figure 9 shows the dual-band working of the proposed design by using the simulated current distribution results obtained from CST. Figure 7, proves that the lower arm only resonates at 3.6 GHz. Whereas in Figure 8, it can be seen that the upper arm only plays a role in achieving the higher resonance frequency, i.e., 4.7 GHz. The location of the two adjacent elements is optimized. Whenever element 1 has its maximum resonance current, element two will have a minimum amplitude of the current, and it can be seen in Figure 9. This arrangement makes the relative currents of the elements orthogonal and results in a low corresponding ECC value (see Figure 6 for ECC results).

**Figure 10:**
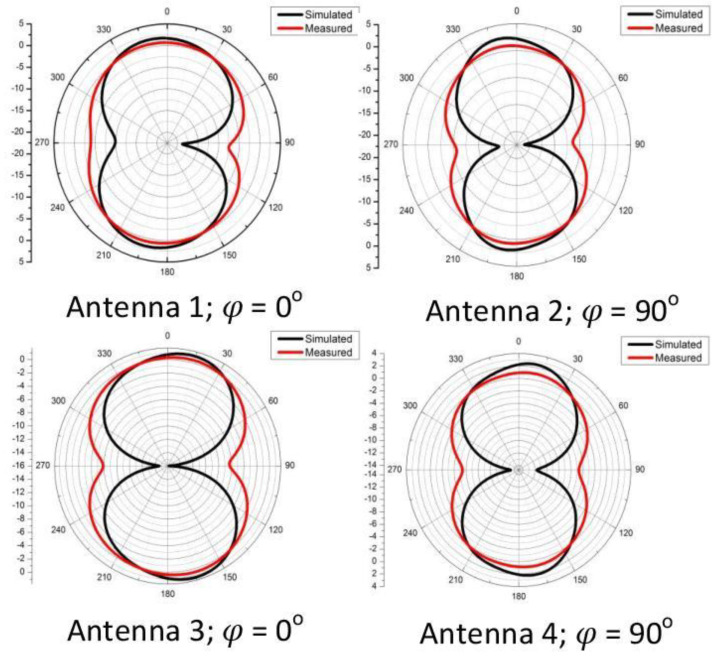
Comparison of simulated and measured radiation pattern of the proposed antenna design at 3.6 GHz.

Figure 10, shows the comparison of measured and simulated radiation patterns. Due to presence of symmetry, we have shown the simulated and measured radiation pattern at 3.6 GHz, in E-plane, i.e., for Antenna 1 and 3, the E-plane is at $\phi =0^{\circ }$, and for Antenna 2 and 4, the E-plane is at $\phi =90^{\circ }$. It is evident from these results that the proposed antenna is working well, as the measured and simulated radiation patterns are in agreement and exhibit the patterns similar to a dipole.

**Figure 11:**
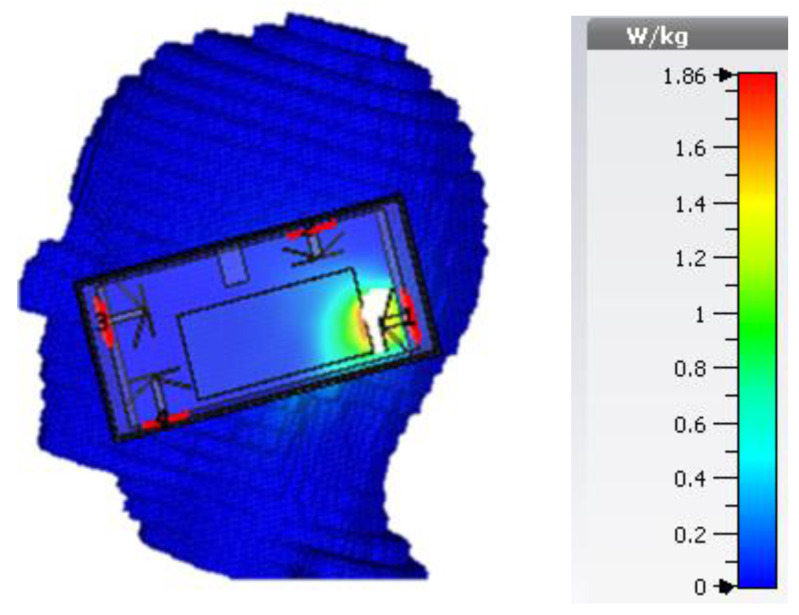
Illustration of Voxel head model incorporating the housing box.

In Figure 11, we have performed the SAR analysis, while incorporating the housing box (for the composition of the housing box, see Figure 3). The SAR values averaged over 10 g of body mass is measured to be 1.8 W/kg at 3.6 GHz and 1.7 W/kg at 4.7 GHz (the maximum input power used for SAR calculation is 0.2 Watts, which in line with the requirements set for the transmission of data in LTE) that is well below the guidelines specified by International Commission on Non-ionizing Radiation Protection (ICNIRP) (i.e., 2.0 W/kg for 10 g) [29]. The small variation in two SAR values can be related to near-field size, which decreases with an increase in frequency.

**Figure 12:**
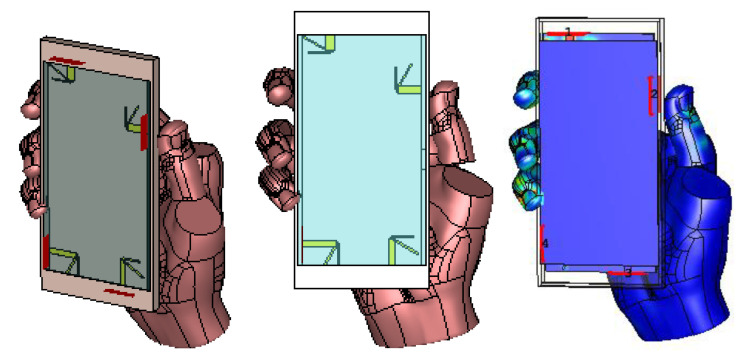
Illustration of the hand model incorporating the housing box.

In Figure 12, we have shown the effect of the human handgrip on the proposed antenna design’s total antenna efficiency. The total antenna efficiency of each element in free space is −2.88 dB (Antenna 1), −3.12 dB (Antenna 2), −1.92 dB (Antenna 3) and −2.45 dB (Antenna 4) at 3.6 GHz, while at 4.7 GHz are −1.61 dB (Antenna 1), −2.19 dB (Antenna 2), −1.72 dB (Antenna 3) and −1.18 dB (Antenna 4), respectively. Moreover, as shown in Figure 12, the effect of the housing box and human handgrip reduces the total antenna efficiency by 5%, as compared to the values calculated in the free space. The small variation in antenna efficiency occurs due to the carefully designed configuration where the elements are placed strategically to minimize the overlap (see Figure 9).

## 5. Conclusions

In this paper, we have proposed a four-element dipole antenna design suitable for 5G-enabled handsets. By utilizing the dual diversity, i.e., radiation pattern and space, the MIMO criteria are satisfied. The proposed antenna is operational in dual sub-6 GHz bands, and the ECC and the loss in capacity between the antenna elements are well below the required threshold. The proposed antenna is fabricated to validate the simulated results. The simulated and measured S-parameters and the radiation pattern results are in good agreement. The measured SAR values averaged over 10 g tissue volume is 1.8 W/kg at 3.6 GHz and 1.7 W/kg at 4.7 GHz, respectively, which satisfy the requirements set by ICNIRP. The proposed antenna’s potential and usability are further validated using the effects of the housing box, and the human handgrip on the antenna efficiency and only a 5% variation is observed. The small variation is due to the carefully designed configuration where the antenna elements are placed strategically to minimize the overlap as much as possible. In the future, we aim to enhance our design to an 8 × 8 MIMO configuration and cover the mmWave spectrum.

## Figures and Tables

**Table 1:** Comparison with the relevant literature.

References	Space Diversity	Pattern Diversity
[10]	✓	✕
[11]	✓	✕
[12]	✓	✕
[13]	✓	✕
[14]	✕	✓
[15]	✓	✕
Proposed antenna	✓	✓

**Table 2:** Design parameters in (mm).

Antenna Element	La	Lb	Lc	Ld	Wa	Wb
Antenna 1	13.47	0.7	13.7	16	4.48	3.22
Antenna 2	13.32	0.7	13.7	16	4.48	3.22
Antenna 3	19.04	1	12.8	17	3.4	2.24
Antenna 4	19.04	1	12.8	17	1.6	3.4

## Data Availability

Not applicable.
